# Co‐Creating Publicly Available Resources to Increase Awareness of and Support for Long Covid Among Ethnic Minority Communities

**DOI:** 10.1111/hex.70596

**Published:** 2026-03-01

**Authors:** Nina Smyth, Ammarah Ahmad, Samina Begum, Ashish Chaudhry, Sophie Clark, Alexa Wright, Karl Gimblett, Damien Ridge, Carolyn A. Chew‐Graham, Dipesh Gopal, Nisreen A. Alwan, Tom Kingstone

**Affiliations:** ^1^ Psychology, School of Social Sciences University of Westminster London UK; ^2^ Advisor member on public and patient advisory group London UK; ^3^ Lower Broughton Health Centre Salford UK; ^4^ Differential Attainment Champion, Salford and Trafford GP Training scheme, NHS England – North West Manchester UK; ^5^ Independent Artist, Emerita Fellow, School of Humanities University of Westminster London UK; ^6^ School of Medicine, Faculty of Medicine and Health Sciences Keele University Keele UK; ^7^ Research & Innovation Department Midland Partnership University NHS Foundation Trust Stafford UK; ^8^ Wolfson Institute of Population Health Queen Mary University London UK; ^9^ School of Primary Care, Population Sciences and Medical Education, Faculty of Medicine University of Southampton Southampton UK; ^10^ University Hospital Southampton NHS Foundation Trust Southampton UK; ^11^ NIHR Applied Research Collaboration Wessex

**Keywords:** art communication, creative methods, ethnic minorities, healthcare, Long Covid, post‐acute COVID‐19 syndrome, primary care

## Abstract

**Introduction:**

Stigma and discrimination make healthcare challenging for people living with Long Covid, especially those from ethnic minority groups. Since their experiences are under‐researched and may differ from other groups, it is crucial that healthcare guidance is informed by the lived experiences of diverse groups.

**Methods:**

Findings from underpinning research (hearing from the unheard: Impact of Long Covid in Black and minority ethnic groups in the UK: HI‐COVE – 31 interviews with ethnic minority individuals living with Long Covid) informed the development of two resources aimed at raising awareness of the challenges faced by ethnic minority groups and offer ways to best support these groups. People living with Long Covid (*N* = 4) provided feedback on the two resources. Feedback was guided by a topic guide. Minimal changes were made following feedback.

**Results:**

Resource 1: Four participants who took part in the underpinning research, worked with an Artist (AW) to curate artwork. The artwork created was a video called ‘Still Looking for Answers’ https://www.youtube.com/watch?v=GDt‐Ro1Cql8&t=1s. It comprises anonymised patient narratives and imagery (performed by actors) and a soundscape to convey ethnic minority lived experiences of Long Covid. Resource 2: an online learning tool called ‘**H**ealth and Social Care **PRO**fessional‐**L**ong **C**ovid’: H‐Pro‐LC tool: https://clineduniverse.org/hicove/story_html5.html shares challenges people from ethnic minority groups face when accessing healthcare for Long Covid. The resource includes guidance on supporting people, particularly people from ethnic minority backgrounds, presenting to primary care with (probable) symptoms of Long Covid.

**Conclusions:**

These publicly available resources aim to raise awareness of Long Covid: they encourage viewers to emotionally connect with experiences of Long Covid as well as offer ways to support people living with the condition, particularly among people from ethnic minority groups.

**Patient and Public Involvement and Engagement:**

The underpinning research of these resources were extensively informed by both patient (*N* = 7) and expert advisory groups (*N* = 6). Co‐creation approaches (through workshops, meetings and written feedback) from people living with Long Covid, carers, stakeholders and members of the public informed the design, development, innovation and impact of resources developed. People with lived experience of Long Covid provided feedback on the resources developed in this study.

## 1 Introduction

Nearly 2 million people in the UK and 200 million people worldwide experience prolonged ill‐health following acute coronavirus disease‐2019 (COVID‐19) [1, 2]. This is known as Long Covid [3] (or post‐acute COVID‐19 syndrome [4]). Symptoms are unpredictable and can be physical, cognitive, or psychological [5] and negatively impact quality‐of‐life [6]. People living with Long Covid experience poor mental health [7] and disruptions to their social and professional lives [6], such as reduced work performance [8].

Long Covid has no current standardised treatment or management approach [9]. Research on patients' experiences of accessing healthcare has highlighted the challenges faced by patients since 2020 [10, 11]. Several years on, patients still report difficulty accessing healthcare, for example, being dismissed by their healthcare provider, and needing to lobby for support [12, 13, 14]. Moreover, patients experience stigma and discrimination within healthcare [15, 16].

Our research (hearing from the unheard: Impact of Long Covid in Black and minority ethinic groups in the UK: HI‐COVE) focused on understanding ethnic minority experiences of accessing healthcare for Long Covid [17] ‐ a group underrepresented in Long Covid research [11, 18] and specialist Long Covid healthcare services [19, 20]. Moreover, current healthcare guidance (e.g. National Institute for Health and Care Excellence: NIHR [21]) draws on patient lived experience. However experiences from ethnic minority groups are crucial to providing quality healthcare for Long Covid [22] and accessible and appropriate care for all [23] as experiences may differ to other groups, yet they are not reflected in the guidance. Our research shows that people from ethnic minority groups experience a lack of symptom awareness of Long Covid, and do not feel a candidate for care [13]. These groups also experience stigma and discrimination which can reduce trust in healthcare and hinder access to appropriate treatment for Long Covid [13]. This can also add trauma for people from these groups accessing healthcare [24]. Emerging research shows the ‘double burden’ accessing healthcare for Long Covid amongst ethnic minority groups, including needing to be persistent to be heard, along with a lack of recognition and empathy from healthcare professionals [25].

Observational data by the Office for National Statistics [26] shows the prevalence of Long Covid is still high, yet healthcare provider support for Long Covid is declining. Increasingly, healthcare professionals may not identify symptoms of Long Covid, as indicated by a decline in the use of the clinical code ‘post‐acute COVID‐19 syndrome’ in primary care [27]. Moreover, Long Covid specialist services [28] differ depending on funding or locality, and some of these services are being disbanded and/or subsumed within other specialist services. Previously, the NHS held a specific website for Long Covid guidance, ‘Your Long Covid Recovery’, however this is no longer available and information on Long Covid is not easily found via the NHS website. Such trends highlight a clear need for continued awareness raising about Long Covid, especially for professionals who support people with symptoms probable of Long Covid (e.g. health or social care professionals, charity representatives, social prescriber link workers). A webtool developed to raise awareness of Long Covid and the stigma associated with it, is the Supporting Long Covid Care (SLCC) tool: https://long‐covid‐care.org.uk. It is aimed primarily at the general public to encourage help‐seeking by people who may have Long Covid with no established diagnosis. It also aims to raise awareness amongst professionals and community workers about the difficulties faced by people with probable Long Covid [29].

In the current study, two resources, informed by underpinning research [13] were developed to raise awareness of Long Covid and available support particularly for people from ethnic minority backgrounds. A video resource utilising art‐based methods (using creative arts to convey lived experiences of health or illness) was developed. Other video resources are available, such as, Healthtalk.org, which provide in‐depth information on topics and experiences relating to Long Covid. The current tool uses art‐based methods to evoke viewers’ imagination, sensory, emotive and cognitive processes [30, 31], to convey complex understandings and meanings of health and connect viewers emotionally with diverse lived experiences and perspectives. Art‐based methods have promoted awareness and knowledge of health conditions, improved health management and reduced inaccuracies about disease and disease prevention [31, 32, 33]. For example, it is useful for increasing awareness of health conditions that are poorly understood or managed [34, 35]. Moreover, artwork can enhance trust when it comes to sensitive health issues by building bridges (e.g. cultural, emotional and intellectual) between researchers and communities, signifying that we are co‐producing and sharing critical stories together [36]. It is particularly useful conveying health issues for diverse groups [37] and enhancing understandings of the cultural and societal issues connected with health, which is critical to addressing the stigma associated with health conditions [31].

A second resource developed was an online learning resource tool to promote essential learning for healthcare professionals about lived experiences of Long Covid amongst people from ethnic minority groups. In healthcare, online large‐scale [38] to smaller [39] resource training is a successful and flexible method of engaging busy healthcare professionals with key learning opportunities [40, 41, 42]. Online Long Covid training is available for healthcare professionals [43] but neglects lived experiences of diverse ethnic groups. Lived experiences enable critical thinking and reflection [44] and promote greater empathy when included in healthcare professional learning tools [45]. Brief online learning resources, offer a flexible way of accessing learning amongst healthcare professionals especially since time and capacity are at a premium.

In the current study, two resources were developed to raise awareness of Long Covid, particularly amongst ethnic minority groups, in addition to providing information on useful avenues of support for these groups. The resources were developed as part of underpinning research (HI‐COVE [13]).

## 2 Methods

The underpinning research [13] is briefly described below, including methods, findings and the patient and public involvement and engagement (PPIE) employed.

### 2.1 Underpinning Research

#### 2.1.1 Methods and Findings

The underpinning research [13] was a qualitative study which explored the lived experiences of ethnic minority individuals living with Long Covid. Semi‐structured interviews were conducted with 15 males and 16 females (aged over 18 years, living in the UK) who were from a range of ethnic minority and socio‐economic backgrounds. Participants were recovered from or living with Long Covid at the time of the interview. Long Covid status was determined by a researcher completing a checklist with participants; this was based on the World Health Organization definition of Long Covid [46]. Topics explored in the interviews were Long Covid status, symptoms, impacts; support and treatment preferences; access to healthcare and wider support systems and networks; challenges to accessing appropriate support. Ethical approval was obtained from the University of Westminster Ethics Committee (ETH2122‐1074). Data were analysed thematically [47] and findings (see Table 1 for a summary) are presented elsewhere [17, 24]. Our expert advisory group met twice, and they informed the data collection and interpretation of interview findings.

**Table 1:** Summary of findings from underpinning research (*HI‐COVE*).

Summary of findings from underpinning research
People living with Long Covid have trouble accessing healthcare e.g. delays in accessing care, needing to lobby for referrals/support. People living with Long Covid reported challenging healthcare interactions e.g. not being believed, being dismissed or not taken seriously. People accessing healthcare experience lack of knowledge, interest and empathy from healthcare providers. Patients from ethnic minority groups experienced stigma & discrimination within healthcare. Participants perceived racism in their healthcare. Community, local and online social links were essential in Long Covid care. Patients valued empathy, validation and fairness when seeking support for Long Covid.

#### 2.1.2 Patient and Public Involvement and Engagement (PPIE)

We embedded the public national standards throughout [48]. People living with Long Covid (1 male and 3 females) informed the development of the underpinning research and its scope for funding acquisition. When conducting the research, a patient advisory group (PAG) informed the research design, recruitment, data interpretation and findings dissemination. The group consisted of 1 male and 6 females living with or caring for someone living with Long Covid, who were from a range of ethnic minority and socioeconomic backgrounds. Two stakeholder workshops (lasting 2.5 h each) were conducted to gain feedback on the preliminary results of the research. The attendees were from different backgrounds (e.g. members from Long Covid charities, healthcare professionals, researchers, people living with Long Covid and people supporting individuals living with Long Covid). Demographic details were not recorded for the workshops. Background status was identified from the details attendees provided for workshop sign‐up. Feedback from attendees was obtained through facilitated discussions and following the event (using a Qualtrics link). Table 2 provides an overview of the workshops, and a summary of the feedback obtained.

**Table 2:** Structure of the online stakeholder workshops and feedback from attendees.

Workshop 1 – held on 18 January 2023	Workshop 2 – held on 23 May 2023
66 attendees	70–80 approx. attendees
**Agenda**	
Overview of HI‐COVE study: aims, PPI, preliminary findings. Talks from guest speakers: – Long Covid Support Group – The Greater Manchester Long Covid Cohort – Lived experiences of Long Covid and recommendations for the recognition, diagnosis and management of Long Covid. Overview of the development of co‐created artwork for Long Covid. Facilitated discussion with participant attendees for each section of workshop.	Overview of HI‐COVE findings: experiences of healthcare access; the need to co‐ordinate own healthcare support; the value of wider systems of support for management of Long Covid. Talks from patients about their lived experiences: – Right Honourable Andrew Gwynne, Labour MP, and Shadow Minister for Health and Social Care ‐ experiences of living with Long Covid, highlighting the importance of a supportive workplace. – HI‐COVE patient advisory group – reflections on being part of Long Covid research. Talks from guest speakers: – Stigma in Long Covid – findings from HI‐COVE study and STIMULATE ICP. – Overview of the use of Long Covid specialist clinics. – Reflections from support groups and people living with Long Covid on navigating care for Long Covid. Showcase video‐artwork: 2 min clip shown to attendees. Facilitated discussion with participant attendees workshop section.
**Reflections from attendees were around the following themes:**	
Underpinning research: – Participant characteristics of the interview study. – Qualitative interview findings. – Reflections on how interview findings resonated with lived experiences. Implications of findings: – The challenges of living with a non‐specific health condition and/or being from an ethnic minority. – The value of sharing lived experiences with others. Development of the video‐artwork: – Initial responses to the artwork were positive. – Suggestions from attendees about content to include in the artwork. – The value of artwork to share experiences and some attendees promoted its wider use. – Ideas for disseminating artwork. Dissemination activities: – Continued and wider raising awareness of Long Covid for the public, healthcare and the government. – Forming a working group for Long Covid learnings and reducing health inequalities.	Underpinning research: – Findings resonated with people's experiences of Long Covid. – Findings resonate with patient stories and healthcare experiences. Lived experience reflections: – Type of support (or lack of) people living with Long Covid have experienced from their workplace. – Healthcare practitioner reflections on how Long Covid patients are typically managed in primary care. – Reflections from people living with Long Covid that it is difficult to seek support from Long Covid specialist clinics. Moreover, there are differences in the support offered depending on location of the clinic due to the services each clinic has on offer. Implications of findings: – More work is needed to support people living with Long Covid. – Need to use findings to continue to raise awareness of Long Covid and/or develop training for health and social care professionals.
See: https://blog.westminster.ac.uk/hicovestudy/study‐updates/ for an overview of the workshops and feedback.	See: https://blog.westminster.ac.uk/hicovestudy/hi‐cove‐stakeholder‐event‐may‐2023/ for an overview of the workshops and feedback.

### 2.2 Development of and Feedback on Resources

The development of the two resources and an overview of the resources are described separately in the following sections. The feedback for the final resources was obtained from four members of the PAG. These are the members of the PAG convened for the underpinning research (as described above). They agreed to provide specific feedback on both resources. Feedback from patient advisors was guided by topics (see Table 3). The lead researcher (NS) collated this via one‐to‐one meetings and/or advisors' written feedback.

**Table 3:** Feedback points on the development of the resources for the patient advisory group.

Resource 1	Resource 2
– Overall impressions of the video‐artwork? – Connections with the themes and experiences conveyed in the video‐artwork? – In what ways (if any) do the themes and experiences conveyed in the video‐artwork impact knowledge about Long Covid?	– In what ways, do the contents of the resource resonate with your experiences of Long Covid? – In what ways, do the experiences expressed in the resource resonate with experiences of being from an ethnic minority group? – What (if anything) is useful about the contents presented in the resource? – Comments about the usability of the resource. – The resource is intended for use by healthcare professionals; however, we want to ensure that the language used, and the content presented is acceptable to patient groups. Please provide any feedback on this.

Feedback from the four patient advisors was organised into four themes: i) overall impressions; ii) knowledge and understandings of Long Covid; iii) connections with the themes and patient narratives conveyed; iv) cultural appropriateness of the content presented in the tool. A description of the resource and patient advisors' feedback are provided separately for each resource. Table 5 provides additional feedback for each theme along with illustrative quotes.

### 2.3 Resource 1: Artwork to Share Lived Experiences of Ethnic Minority Individuals Living With Long Covid

#### 2.3.1 Development Process for Creating the Artwork

An aim of the underpinning research was to work with an Artist to co‐create artwork to share lived experiences of ethnic minority individuals living with Long Covid. The Artist (AW: a member of the research team), female and white ethnicity, works mainly with lens‐based media and installation. Her work typically involves collaboration with healthcare professionals and working closely with people with poor mental or physical health. She engages with people with lived experience through a participatory process that facilitates personal storytelling, and participants are invited to explore ways of communicating their lived experiences using visual media. Her work aims to empathetically address challenges to identity, or wellbeing in a way that is beneficial to participants, as well as being accessible to broad audiences. Her work is sensitive to social contexts where people feel marginalised or vulnerable.

The Artist worked with selected participants who took part in an interview for the underpinning research. At the end of each interview, participants were informed about the artwork development and were sent an information sheet. Interested participants completed a consent form, agreeing to be contacted by the Artist and for their anonymised interview transcript to be shared with, and reviewed by, the Artist. The Artist selected which participants to invite to be involved in the co‐creation of the artwork, and this was based on key themes. The themes were from the underpinning research. Selection was also made to include male and female participants and participants from a range of ethnic backgrounds.

Four participants contributed to the co‐creation of the artwork: one male and three females: one of mixed heritage, one from a Black background, and two from a South Asian background. To maintain anonymity of participants, individual demographic information was not provided.

The Artist attended research team meetings to be familiar with the interview topics, data analysis and interpretation. The Artist also discussed with the research team the core themes that would be conveyed in the artwork; these core themes were determined by data analysis of the underpinning research and conversations with the four participants involved in the artwork creation.

During the production process the Artist met individually with the four participants, two to four meetings were held in‐person (the Artist travelled to participants' chosen location). During the first meeting, participants were asked to elaborate on their original interview to enhance the Artist's understanding and interpretations of their lived experiences. Subsequent conversations focused on imagery that might best represent participants' lived experiences for the artwork. With permission, conversations were recorded and transcribed (verbatim), or detailed notes were made by the Artist.

At each stage, participants were able to edit transcripts and approve them. The Artist discussed possible mediums for the artwork with participants. Drawing on the Artist's background, a video was chosen as this is a portable and accessible medium, and one where spoken narrative can be integrated with visual images. Spoken narratives were derived from what the four participants said. The Artist edited these to distil the sections relevant to the study aims and preserve participant anonymity. Actors were chosen to closely represent the participants involved in the co‐creation of the video‐artwork. One person portrayed in the video‐artwork was a participant involved in the co‐creation of the video‐artwork; the participant felt strongly that they wanted to perform their own narrative. The participants' gender and ethnicity are portrayed in the video, with consent for the final version of the video‐artwork being published.

The emerging artwork was shared with the four participants, providing an opportunity for participants to give feedback and contribute their ideas, and for the Artist to work responsively to participants' suggestions. Participants were offered a £35 voucher as a token of appreciation for each meeting. As the artwork was developed it was shared at the two online stakeholder meetings (see Table 2 for feedback). The research team reviewed the emerging artwork to provide feedback and ensure fidelity with the analysis conducted as part of the underpinning research. Participants agreed in writing to the video content and its publication. Participants were all highly invested in the work, and two were especially connected with the videos, and became visibly emotional, as they felt this was the first time they were able to communicate their experiences in a way that was widely understandable.

The final artwork: ‘Still Looking for Answers – the unheard voices of Long Covid’.

The final artwork is a video comprising still and moving images, driven by participant narratives, and participatory activity, and a commissioned soundscape plays throughout. The video‐artwork is freely available, and a shorter version is made available for easy sharing (see Table 4 for hyperlink).

**Table 4:** Hyperlinks for the two resources developed.

**Resource 1: ‘Still Looking for Answers’** https://www.youtube.com/watch?v=GDt‐Ro1Cql8&t=1s **Short version** https://www.youtube.com/watch?v=hyERC7CF0ac
**Resource 2: Health and Social Care PROfessional‐Long Covid (H‐Pro‐LC) tool** https://clineduniverse.org/hicove/story_html5.html

The video presents Long Covid narratives, interspersed with landscape imagery and participatory activities to convey lived experiences that are difficult to articulate. All narrative and participatory activity is performed by actors. Themes from the underpinning research are conveyed throughout: What Long Covid is and how it impacts daily lives, relationships and work, and the experiences of accessing healthcare and wider support. Anonymised personalised experiences are expressed from the four participants, to encourage viewers to connect emotionally with various Long Covid realities.

**Figure 1:**
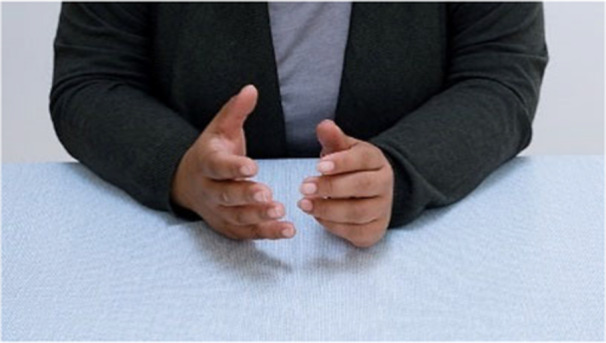
Screenshot of the video of participant narrative conveyed in the video‐artwork (resource 1).

**Figure 2:**
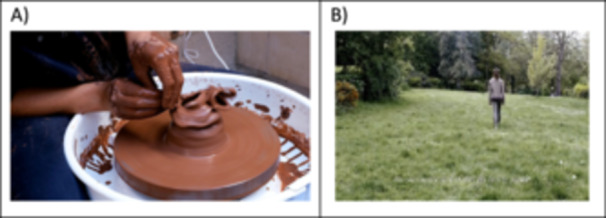
Still images from video‐artwork (resource 1). (A) The image shows the hands of a potter throwing a vessel on a wheel that repeatedly collapses. This was suggested by one of the participants to represent their frustrated efforts in attempting to achieve things during their experiences of living with Long Covid. (B) Image shows landscape chosen to convey a metaphor for internal, emotional states of Long Covid.

Persons, portrayed in the videos to share individual stories of Long Covid, are faceless with only hands and upper body visible. This was chosen to retain participant anonymity and convey expression using hands and body language (see Figure 1). Showing only the hands or upper body leaves the identity of the participants open to the viewers' imagination and potentially enables more people to identify with the narrators. The still and moving imagery presented in the video‐artwork were suggested by the four participants. Imagery includes both landscape and/or participatory activity (see Figure 2). Quotes from the interviews conducted as part of the underpinning research are presented throughout the video‐artwork, these are presented to represent broader spectrums of Long Covid realities. For some of the quotes these are spoken in different languages to represent the diversity of participant languages. A bespoke sound piece is portrayed throughout to reinforce the atmosphere of the visual imagery. The work was commissioned by a sound composer (Ben Macdonald).

### 2.4 Feedback on the Development of the Video‐Artwork From Patient Advisors

#### 2.4.1 Overall Impressions of the Video‐Artwork

Overall feedback from the patient advisors was positive and they suggested minimal changes

The video‐artwork is really well put together and sadly (but accurately) captures the dysfunction of Long Covid paired with the emotional roller‐coaster that it is. The images and short videos used, neatly correspond to the different types of despair that are being described.

(Patient 4)

#### 2.4.2 Knowledge and Understandings of Long Covid

Patient advisors reflected on how Long Covid realities conveyed in the video‐artwork resonated with their lived experiences of Long Covid. They felt the video‐artwork captured different themes (i) understanding of what Long Covid is; (ii) the struggles faced by people living with Long Covid (Q1); (iii) challenging healthcare encounters e.g. not being understood, being dismissed, experiencing discrimination (Q2); (iv) the value of support groups for Long Covid (Q3); and (v) and the impact of being ill and how life changing it is – a loss of identity (Q4), the impact mentally, socially, and in the workplace (Q5). Table 5 presents additional feedback from the patient advisory group.

**Table 5:** Feedback points on the development of the resources from the four patient advisors.

Feedback theme	Resource 1: video‐artwork	Resource 2: online learning tool
Overall impressions		**Q1:** ‘The toolkit was in an easy‐to‐read format’. (Patient‐3) **Q2:** ‘I like how interactive the resource is, very good navigation design’. (Patient‐1) **Q3:** ‘Thought the speech was quite slow, but that was almost emphasising the condition and encouraged the viewer to reflect on stories.’ (Patient‐2)
Knowledge and understandings of Long Covid	**Q1:** ‘Not knowing who you are anymore and learning to live a new way of life and figuring out who you are now’. (Patient‐1) **Q2:** ‘[the artwork portrays] perceptions of discrimination and awareness of the challenges to accessing healthcare'. (Patient‐2) **Q3:** ‘I remember the support that I found in support groups’. (Patient‐2) **Q4:** ‘Losing of one's Identity, Long Covid is what defines you’. (Patient‐3) **Q5:** ‘Disability in the workplace such an important issue covered because so many people have lost their lost their job, like myself’. (Patient‐1).	**Q4:** ‘This is a very comprehensive tool; this reminds me of the tools developed to support domestic violence advocates to complete the assessment forms for victims and the training tools provided to do this…I feel [this is] a great informative tool for professionals to use to learn about [Long] Covid. I am just comparing the quality of the information and the tools developed because it shows to me how much work has been put into this, to be [a] national benchmark for the Long Covid community to be used just like the domestic abuse tools developed and used in the women's sector'. (Patient‐1)
Connections with the themes and patient narratives conveyed	**Q6:** ‘Many of my physical symptoms have improved and I had forgotten about them, this was a reminder of the physical symptoms, the disability…and the dependency on family and my wife. I have forgotten about my post exertional fatigue and brain fog which lasted most of 2020'. (Patient‐2) **Q7:** ‘Even today these experiences I can resonate with because when I get a cold I have similar symptoms as before’. (Patient‐1) **Q8:** ‘…It [video‐artwork] validated and encapsulated many of my emotions [I felt] at the time.’ (Patient‐2)	**Q6:** ‘Unfortunately, I don't think there is any section that I haven't felt, experienced or personally thought about.’ (Patient‐4) **Q7:** ‘[the] stories could have been me or anyone else with Long Covid‐ whilst the images were that of ethnic minorities, the symptoms could have been experienced by anyone, regardless of ethnicity’. (Patient‐2)
Cultural appropriateness of the content presented in the tool	**Q9: ‘**The stories told by my people, each one has their own importance… The experiences told by people I can relate to.’ (Patient 1)	**Q8:** ‘The images of people being diverse along with the accents and voices of individuals conveyed the array of experience of Long Covid from perspectives of being from an ethnic minority group’. (Patient‐1)

#### 2.4.3 Connections With the Themes and Patient Narratives Conveyed

All patient advisors reflected on the emotional connections they experienced watching the video‐artwork:

Some parts of this [video‐artwork] have made me emotional, reminded me of the hard struggles and the word independence from one of the testimonials hit hard, because we all want to be independent and not want others to help us due to ill health. It's been a horrible journey. Brought tears to my eyes.

(Patient 1)

It reminded them of the trauma they experienced at the time of experiencing Long Covid symptoms:

The video brought out emotions I had experienced before. Bring[ing] in a void, loss of hope, loss of normality, loneliness and denial by myself and those around me. Not knowing what was going on.

(Patient 2)

The video was like they were ‘re‐living’ their struggles, symptoms or emotions experienced (Q6, Q7). They also reflected on a variety of positive emotions they experienced when viewing the video‐artwork, such as feeling validated (Q8), hope, not feeling alone, and being believed:

This video makes me feel like I am not alone in late 2024 meaning in the present time because I have not recovered and still have Long Covid symptoms…I feel a sense of calmness watching the video overall and hope.

(Patient 1)

Creative art methods displayed in the video helped patient advisors connect emotionally with the experiences and themes portrayed. For instance, showing only a person's body and hands (Figure 1) helped them form emotional connections with the Long Covid realities conveyed:

I like how faces are not shown; in a way this shows how Long Covid survivors have been pushed aside like we don't exist and how we have been left to our own accord to recover.

(Patient 1)

Only showing people's hands has all worked really well. Almost emphasising the hidden voices [of people living with Long Covid].

(Patient 3)

Emotional connections were made with the still and moving imagery. For instance, the moving imagery of collapsing pottery (see Figure 2a) reminded a patient advisor of the many challenges of Long Covid:

The image of the pot being built up and down, and being refreshed and spinning round and round resonated with me. Almost reminding me of the trials and challenges, and groundhog day nature of the condition.

(Patient 2)

Another patient advisor commented on the connections made with Figure 2b showing a person walking through landscape; this provided a reminder of the physical struggles of Long Covid and the uncertainty of recovery:

I like how there is a person just walking because it reminds me of struggles of walking again and having some baseline but also this feeling of the never‐ending struggles of not knowing when I will be fully recovered, walking with no destination.

(Patient 1)

A bespoke soundscape is played throughout. A patient advisor commented that the music goes from low to high, which adds a ‘dramatic effect’ and ‘gives a cultural context’.

#### 2.4.4 Cultural Appropriateness of the Content Presented in the Tool

Patient advisors felt the lived experiences of ethnic minority individuals living with Long Covid is conveyed throughout and they liked how they were told from the perspective of ethnic minority individuals (Q9):

Discrimination is cross cutting theme in accessing healthcare.

(Patient 3)

### 2.5 Resource 2: An Online Learning Tool Promoting Essential Learning for Healthcare Professionals, About Lived Experiences of Ethnic Minority Individuals Living With Long Covid

#### 2.5.1 Development Process for Creating the Online Learning Tool

As part of the stakeholder workshops (described above) training around Long Covid for healthcare professionals was considered a key area for future work. The tool was developed to provide informational resources for healthcare professionals to support patients entering primary care for (probable) symptoms of Long Covid. It was designed to be accessible for wider groups who support people living with the condition (e.g. social care professionals, carers, social prescriber link workers).

The tool was developed, based on the underpinning research [13]. The content of the tool was initially devised by authors NS, TK and KG, they designed the format and structure of content presented. All authors have experience developing guidance or learning resources for communicating ways to support health. For example, author NS developed guidance for self‐harm [49]; author TK developed learning resources on mental‐physical co‐morbidity for healthcare professionals [50, 51] and research methods [38, 52]; author KG develops teaching material for postgraduate clinicians. The wider team have expertise developing online training resources for healthcare professionals. For example, author CCG designed the Royal College of General Practitioners (RCGP) e‐learning for Long Covid [53] and author NA led development of the SLCC tool [29].

**Figure 3:**
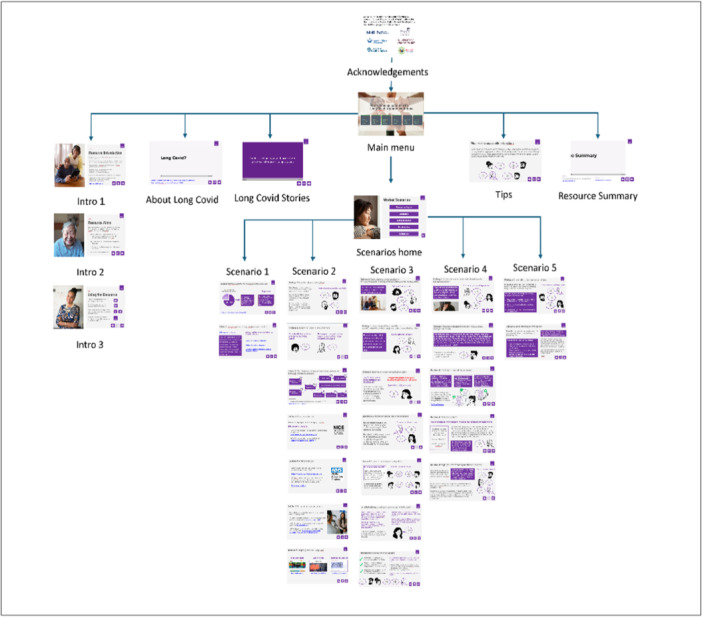
Structure of online tool (resource 2).

A provisional outline structure was designed along with a list of intended learning outcomes, by author KG. The outline was intended to reflect core themes from the underpinning research [17, 24] and promote essential learning for healthcare professionals. The outline was further refined through discussion within the multi‐disciplinary team authors DR, CCG, DG and NA. Though the content of the tool was not informed specifically by the patient advisors, the content reflects the core themes that emerged from the underpinning research, and the patient advisors were involved in the data interpretation and theme development. Feedback was obtained from the patient advisors on the near final version of the tool. The final structure is outlined in Figure 3.

**Figure 4:**
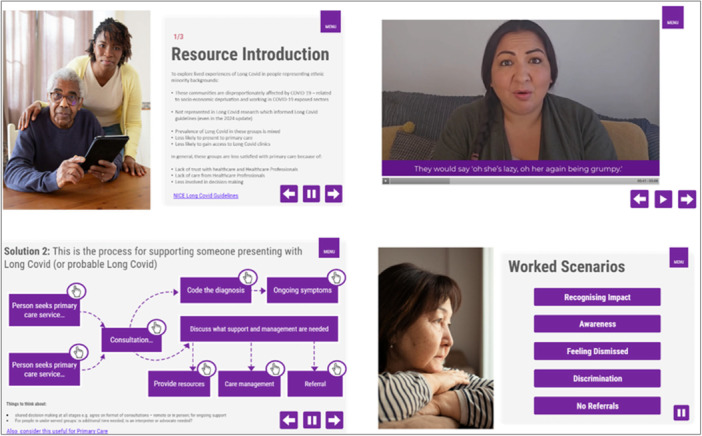
Screen content for the online tool (resource 2).

The tool content was embedded into PowerPoint slides and then transferred to an e‐learning authoring application called *Articulate Storyline*. *Storyline* was used to create the underlying platform from which the learning content is presented to learners. The content was created using: *TechSmith Camtasia* (video editing); *Adobe Photoshop* (image editing); *Audacity* (audio editor); and *Handbrake* (video resizing application). An example of typical screen contents and layouts are shown in Figure 4.

#### 2.5.2 The Final Online Learning Tool: The **H**ealth and Social Care **PRO**fessional‐**L**ong **C**ovid (H‐Pro‐LC) Tool

The tool is presented using different formats, including, written, visual, audio and video elements. Signposting and hyperlinks to existing resources were included. Qualitative data extracts were anonymised quotes, not original audio recordings, from the underpinning research. These were read by actors for video recordings and recorded to support user interactivity and engagement. Demographic information provided by participants was used to match voiceover artists to maintain a sense of authenticity. The tool begins with an introduction and ends with a summary of key take home messages. The main section of the tool is split into four sections.

Section 1 provides information and learning on Long Covid, in the form of statistics, definitions and guidance as well as signposting to external articles and resources, appropriately referenced throughout to support understanding of Long Covid. This was identified as contributing to patients experiencing challenging healthcare encounters explored in the underpinning research.

Section 2 presents stories of Long Covid; these are constructed based on common experiences of the participants interviewed as part of the underpinning research. Patient stories conveyed lived experiences of Long Covid to enhance understanding of the challenges ethnic minority people may face since a lack of understanding and empathy contributed to negative healthcare encounters.

Section 3 presents worked scenarios to provide examples of the different challenges people face when accessing care: key themes from the underpinning research which contributed to patients' negative healthcare encounters were (i) lack of recognition of the suffering caused by Long Covid; (ii) lack of awareness of Long Covid; (iii) feeling dismissed; (iv) experiencing discrimination; (v) difficulty accessing specialist or onward care. An overview of the challenges are presented along with quotes from participants to illustrate the challenges and associated impact. Solutions are presented after each challenge ‐ guidance and information are provided. They are intended to support healthcare professionals supporting people presenting with Long Covid symptoms.

Section 4 presents top tips for practice – sharing patients' desires when seeking healthcare support. The resource ends with a summary highlighting the key take home messages that are pertinent to delivering better healthcare for ethnic minority groups.

### 2.6 Feedback on the Development of the Video‐Artwork From Patient Advisors

#### 2.6.1 Overall Impressions of the Tool

Patient advisors thought the tool provided information resources and patient stories to convey understanding about Long Covid:

There's a really good balance of personal perspectives and helpful resources with findings of the study central to this resource. I'm highlighting this as although this is the point of the resource the findings and common themes could have easily been lost, and it hasn't.

(Patient 4)

Overall, there was positive feedback on the usability and presentation of the tool. The tool was seen as straightforward to use, although, it may take a little time to navigate at first (Q1). Patient advisors considered it ‘engaging’ and thought the use of different methods to share and/or convey information (e.g. videos, audio, written text, speech bubbles, statistics, hyperlinks, diagrams, graphics) was helpful to absorb information (Q2). Moreover, they reported that the speech was pitched at the appropriate speed (Q3). Patient advisors suggested minimal changes but commented that some images may not reflect content presented in the audio clips and written text. We used alternative images to ensure they accurately reflected what was being conveyed.

#### 2.6.2 Knowledge and Understandings of Long Covid

The information and resources provided in the tool (Section 1) were felt by patient advisors to be informative for patients and professionals, and that a wealth of quality information is provided (Q4). It was thought to serve as a good informational tool to help GPs navigate the nuances of Long Covid, helping them support their patients.

In Section 3, the scenarios presented were considered useful. For instance, the scenario related to communicating with patients was seen as useful to prompt healthcare professionals to consider how to maximise use of their consultations. As such, this can be used as a communication guide for Long Covid consultations for junior doctors and GPs alike:

[The] scenario section is super great, so many people will have questions [and] challenges and not knowing how to resolve them and this gives a clear outline of how to do this.

(Patient 1)

In Section 4, a schematic diagram is presented to help healthcare professionals navigate Long Covid care; one patient advisor felt that this would help healthcare professionals support their patients on their Long Covid journey. However, one felt more emphasis was needed that Long Covid clinics are no longer available and healthcare professionals must actively find local support. More details on the referral pathway were added. The reflective activities were thought to be useful for healthcare professionals:

I liked the reflective activity slide. This will help professionals challenge their own view behaviours towards Covid.

(Patient 1)

#### 2.6.3 Connections With the Themes and Patient Narratives Conveyed

Patient advisors resonated with the experiences of living with Long Covid conveyed throughout the tool (Q6). Notably, they felt the tool provided validation of their illness and experiences:

All the content [of the tool] in one way or another I relate to because I have been exposed to it, experienced it and are the challenges I have faced. It really captures the experience of a Long Covid Patient.

(Patient 1)

Patient advisors thought the stories powerfully conveyed their perspectives (Q7). Not all Long Covid patients will experience typical or common Long Covid symptoms; a patient advisor felt this was an important aspect of the patient stories ‐ the *‘hidden illnesses’* amongst Long Covid sufferers. A key theme portrayed throughout the patient stories was the functional impact of Long Covid on the patients, relatives, and social structures which may encourage healthcare professionals to explore this in their consultations. Patient advisors felt these stories conveyed the importance of being believed and validated:

[the tool] resonates [with me], particularly the emphasis of believing the person in front of you and not discounting their previous experiences with the other healthcare providers.

(Patient 4)

The illustrations used throughout were viewed positively and added an emotional connection for viewers:

The illustrations in animation were powerful messages that brought to the forefront an emotional and painstaking journey.

(Patient 2)

#### 2.6.4 Cultural Appropriateness of the Content Presented in the Tool

Patient advisors felt the tool accurately reflected experiences of Long Covid in a culturally sensitive manner:

I feel like you have really captured the experience of Long Covid patients. You have been very mindful of any sensitive/incorrect words being used.  Especially anything which can be misinterpreted. Sensitive to different cultures as well.

(Patient 1)

They also felt the images and voices used were diverse (Q8). The underpinning research explored experiences of Long Covid from people from different ethnic minority backgounds. The tool uses audio and video clips including Long Covid stories from people from diverse ethnic backgrounds. A patient advisor felt that this helps the viewer feel like they are not the only one suffering from Long Covid as well as conveying that Long Covid can affect anyone. Through the testimonials of people shown in the tool they reflect the experiences from an ethnic minority person who has Long Covid; they show some of the challenges for example, discrimination, emotions, day to day struggles, lack of support, and not wanting to ask for or access support:

Perceptions of discrimination and awareness that challenge me [to] access healthcare.

(Patient 2)

Quotes from the interviews are presented throughout and a patient advisor reflected on the use of these short quotes as adding to the impact conveyed throughout the video. Short sentences spoken in different languages are also presented; a patient advisor felt the use of dialogue in different languages also helped them connect with people's suffering and different communities by making them feel that they are not alone:

The subtitles in the start along with the different languages, even though I didn't understand the language, [they] made me focus more on the words and understanding the emotional pain that was being expressed.

(Patient 3)

## 3 Conclusions, Future Engagement and Impact

Two resources were curated from underpinning research [13] which focused on understanding the lived experiences of Long Covid amongst people from ethnic minority backgrounds [17, 24]. The aim of developing and sharing these resources was to raise awareness of lived experiences of ethnic minority individuals living with Long Covid in addition to providing information on useful avenues of support. The resources curated included a piece of video‐artwork (resource 1) and an online learning tool (resource 2). We consulted members of our PAG on the development of these resources. We also obtained feedback from patient advisors (who were part of the PAG) on the final content and presentation of both resources. Patient advisors provided positive feedback on both resources and suggested minimal changes. Additionally, attendees at the stakeholder workshops, conducted as part of the underpinning research, provided feedback on the video‐artwork (resource 1) and discussions held at these workshops fed into the development of the H‐Pro‐LC tool (resource 2). Both the PAG and attendees at the stakeholder workshops promoted the development of these resources. They felt more work is needed using creative methods, e.g. artwork as a medium to raise awareness of health research.

The video‐artwork was co‐created with ethnic minority people living with Long Covid and an Artist with a background in developing visual works to communicate the lived experiences of health through storytelling using visual media. The artwork was designed to promote engagement and interaction from the public about lived experiences of ethnic minority individuals living with Long Covid. We used art as a medium to share lived experiences of Long Covid as this condition is poorly understood and associated with stigma [15, 16, 54, 55, 56, 57]. Furthermore, this representation of anonymised individualised experiences of Long Covid, encourages viewers of the artwork to emotionally connect with experiences of illness. This helps enhance trust and empathy of others [36] and reduce stigma associated with sensitive health conditions [31] and/or experiences of marginalised groups [37].

Art‐health communication is most effective when the Artist co‐creates with people with lived experiences, researchers and healthcare professionals [58]. As such, the artwork was co‐created with people with lived experience of Long Covid, thus providing a medium to understand, interpret and communicate experiences in a therapeutic and/or cathartic manner. Involving people with lived experience in the creation process, facilitated by a professional Artist, maintains authenticity to the piece and the expressed messages [59, 60]. Patient advisors felt that the themes presented in the video‐artwork resonated with their Long Covid realities and that of others. All the patient advisors reported emotional connections with the themes presented in the video‐artwork. Moreover, for participants involved in the creation of the artwork it provided therapeutic benefits by allowing them to express their experiences in different ways.

During development of the video‐artwork, the research team expressed a preference for the themes presented throughout the artwork to complement and be underpinned by the qualitative analysis conducted as part of the underpinning research [13]; ensuring the shared experiences of the participants were represented appropriately. Qualitative rigour and the artistic process were negotiated throughout. On reflection, the freedom of expression may have inadvertently been constrained and overshadowed by qualitative rigour with some themes identified as part of the one‐to‐one discussions with the artist not being portrayed in the final artwork.

The H‐Pro‐LC tool (resource 2) shares some of the challenges ethnic minority people face when accessing healthcare. The tool provides information and signposts guidance on ways to support healthcare interactions, primarily designed for healthcare professionals since guidance on Long Covid is limited and does not represent marginalised groups [21]. This was further identified as a key need from the stakeholder workshops held as part of the underpinning research [13]. The H‐Pro‐LC tool complements the SLCC tool [29]; both tools are aimed at raising awareness and understanding of Long Covid realities for people with (probable) symptoms of Long Covid and health and social care professionals. The H‐Pro‐LC tool is an interactive learning tool aimed at signposting guidance on ways to support Long Covid for health and social care professionals. The SLCC tool is primarily aimed at encouraging people with (probable) Long Covid to seek healthcare for support.

Feedback from the PAG was positive; they viewed the H‐Pro‐LC tool as useful in connecting the viewer (both patients and healthcare professionals) with Long Covid realities and it offers key learnings of Long Covid. However, a patient advisor felt that the cultural competency could be enhanced further by encouraging healthcare professionals to think about the approaches they use when they engage with people from different backgrounds in receiving a diagnosis and treatments.

Resources are freely available, accessible by the public and health and social care professionals (see Table 4 for resource hyperlinks). Future work will track engagement with the resources and explore ways in which the resources enhance viewers' or users' awareness of Long Covid, and ways to support people living with Long Covid. Specifically for the video‐artwork, we aim to share it with people with lived experience of Long Covid, people who provide both formal and informal care for people with Long Covid, and members of the public. Feedback will be obtained on how viewers connect with the narratives portrayed in the video‐artwork and how it impacts their understandings and perceptions of Long Covid. For the online learning resource tool, we seek to share the resource with formal carers of people living with Long Covid, such as, members from charities and healthcare professionals. Feedback will be obtained on ways the resource was used in providing care to people presenting to healthcare with probable Long Covid symptoms. Whilst the tool is publicly available, we will continue to update the informational resources presented in the tool periodically to ensure guidance is kept up to date.

The resources are intended to raise awareness of the lived experiences of people living with Long Covid who are from an ethnic minority background however the experiences conveyed, and informational resources provided may also be relevant and useful to other underrepresented communities and/or other stigmatised or less well understood health conditions. A strength of the work is the diversity of experiences of Long Covid conveyed throughout the two resources. The video‐artwork was co‐created and widely informed by patients, people who support people living with Long Covid, and members of the public. The online learning tool was developed in response to discussions with attendees from the stakeholder groups to ensure action is taken to support people living with Long Covid. Although positive feedback from patient advisors has been obtained it is essential to share the resources widely and to gain further feedback from more diverse groups, including the public and professionals working with ethnic minority individuals who may have symptoms of Long Covid.

## Author Contributions

**Nina Smyth:** conceptualisation, investigation, funding acquisition, writing – original draft, methodology, writing – review and editing, formal analysis, project administration, data curation. **Ammarah Ahmad:** methodology, writing – review and editing. **Samina Begum:** methodology, writing – review and editing. **Ashish Chaudhry:** methodology, writing – review and editing. **Sophie Clark:** methodology, writing – review and editing. **Alexa Wright:** conceptualisation, investigation, funding acquisition, methodology, writing – review and editing. **Karl Gimblett:** methodology, writing – review and editing. **Damien Ridge:** conceptualisation, investigation, funding acquisition, methodology, writing – review and editing; formal analysis, project administration, supervision, data curation. **Carolyn A. Chew‐Graham:** conceptualisation, investigation, funding acquisition, writing – review and editing, methodology, formal analysis, project administration, supervision, data curation. **Dipesh Gopal:** conceptualisation, investigation, funding acquisition, methodology, writing – review and editing, data curation. **Nisreen A. Alwan:** conceptualisation, funding acquisition, writing – review and editing. **Tom Kingstone:** conceptualisation, investigation, funding acquisition, methodology, writing – review and editing, data curation.

## Ethics Statement

Ethical approval was obtained from the University of Westminster Ethics Committee (ETH2122‐1074).

## Conflicts of Interest

NA is co‐investigator on NIHR‐funded STIMULATE ICP study and has contributed in an advisory capacity to the World Health Organization and the European Union Commission's Expert Panel on effective ways of investing in health meetings in relation to post‐COVID‐19 condition. CCG is a co‐investigator for NIHR grant, ‘The PHARM‐LC Study: What role can community PHARMacy play in the support of people with Long Covid?’, and in receipt of NIHR funding for, ‘Symptom patterns and life with post‐acute COVID‐19 in children and young people: SPLaT‐19 cohort and qualitative study’. CCG has received Honoraria for delivering training to GP trainees on Long Covid via Health Education England. DR was a co‐investigator on the Roche funded study, ‘Investigating how carers cope, access and use support services – Lessons from Covid‐19, Portraits of Care’.

## Data Availability

The authors have nothing to report.
